# Progress, Challenge, and Prospect of LiMnO_2_: An Adventure toward High‐Energy and Low‐Cost Li‐Ion Batteries

**DOI:** 10.1002/advs.202304938

**Published:** 2023-11-14

**Authors:** Jin Ma, Tingting Liu, Jie Ma, Chi Zhang, Jinhu Yang

**Affiliations:** ^1^ Research Center for Translational Medicine East Hospital Tongji University School of Medicine No. 150 Jimo Road Shanghai 200092 P. R. China; ^2^ School of Chemical Science and Engineering Tongji University Siping Road 1239 Shanghai 200092 P. R. China; ^3^ Research Center for Environmental Functional Materials State Key Laboratory of Pollution Control and Resource Reuse College of Environmental Science and Engineering Tongji University 1239 Siping Road Shanghai 200092 P. R. China

**Keywords:** element doping, Li‐ion batteries, LiMnO_2_ cathode, surface coating, synthesis methods

## Abstract

Lithium manganese oxides are considered as promising cathodes for lithium‐ion batteries due to their low cost and available resources. Layered LiMnO_2_ with orthorhombic or monoclinic structure has attracted tremendous interest thanks to its ultrahigh theoretical capacity (285 mAh g^−1^) that almost doubles that of commercialized spinel LiMn_2_O_4_ (148 mAh g^−1^). However, LiMnO_2_ undergoes phase transition to spinel upon cycling cause by the Jahn‐Teller effect of the high‐spin Mn^3+^. In addition, soluble Mn^2+^ generates from the disproportionation of Mn^3+^ and oxygen release during electrochemical processes may cause poor cycle performance. To address the critical issues, tremendous efforts have been made. This paper provides a general review of layered LiMnO_2_ materials including their crystal structures, synthesis methods, structural/elemental modifications, and electrochemical performance. In brief, first the crystal structures of LiMnO_2_ and synthetic methods have been summarized. Subsequently, modification strategies for improving electrochemical performance are comprehensively reviewed, including element doping to suppress its phase transition, surface coating to resist manganese dissolution into the electrolyte and impede surface reactions, designing LiMnO_2_ composites to improve electronic conductivity and Li^+^ diffusion, and finding compatible electrolytes to enhance safety. At last, future efforts on the research frontier and practical application of LiMnO_2_ have been discussed.

## Graphical abstract

A LiMnO_2_ cathode with high theoretical capacity shows great commercial value due to its low price and nontoxicity. This review summarizes the overall progress and challenges of LiMnO_2_, focusing on crystallographic structures, synthesis and modifications like element doping, surface coating, composites designing, and electrolytes matching for electrochemical performance improvement. Future perspectives on the research frontier and practical application are also discussed.

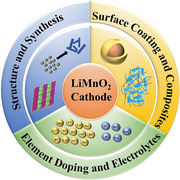

## 1 Introduction

Over the past few decades, significant efforts have been devoted to developing advanced energy storage devices and new renewable energy sources to cope with the fossil energy crisis and environmental problems. Since the C║LiCoO_2_ battery was commercialized by Sony in 1991,^[^ ^1^ ^]^ lithium‐ion batteries (LIBs) have been used in a wide range of portable electronic devices, hybrid and full electric vehicles and other energy‐storing devices because of their high energy density, long cyclability, low self‐discharge and absence of memory effect.^[^ ^2^, ^3^ ^]^ Reducing greenhouse gas emission has now become global consensus in response to the greenhouse effect. For example, in October 2021 the Chinese government has proposed the goals of peak carbon dioxide emissions and carbon neutrality which will be achieved in 2030 and 2060, respectively. In this context, there is no doubt that the new energy technology is inevitable to achieve carbon peak and carbon neutrality. Thus, as a representative of clean energy storage devices, LIBs stand for an ideal candidate due to high energy density, low cost, safety, and environmental sustainability, which are of particular importance.

**Figure 1:**
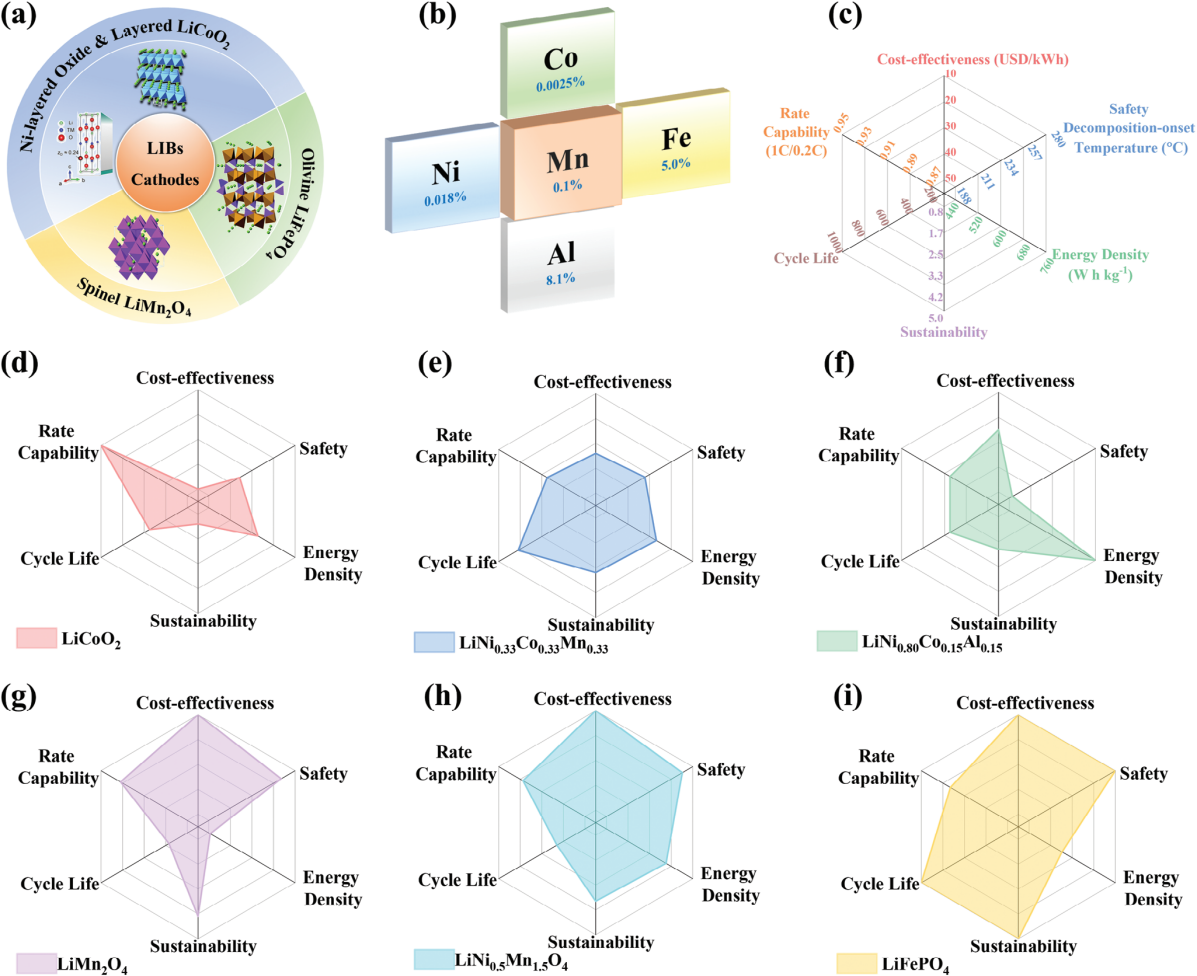
a) Three main types of cathode materials commercialized for lithium‐ion batteries. Reproduced under terms of the CC‐BY license.^[^ ^7^ ^]^ Copyright 2014, M. Saiful Islam and Craig A. J. Fisher, published by Royal Society of Chemistry. Reproduced with permission.^[^ ^8^ ^]^ Copyright 2017, American Chemical Society. b) Crustal content (marked in blue font) of metal element involved in commercialized cathode materials for LIBs. c) Radar maps for cathode materials commercialized for lithium‐ion batteries.

LIBs are mainly made of cathode, separator, electrolyte and anode, where the cathode limits the energy density and dominates the final cost of the battery.^[^ ^4^, ^5^, ^6^ ^]^ Up to now, three main types of cathode materials have been commercialized, including layered lithium transition metal oxide LiTMO_2_ (TM = Co, Ni, Mn), spinel LiMn_2_O_4_, and olivine‐structure lithium iron phosphate LiFePO_4_ (**Figure** **1**a).^[^ ^7^, ^8^ ^]^ In terms of cathode materials, metal elements play an essential role in the advancement of energy storage. As shown in Figure 1b, the crustal content of common metals is provided, including nickel (Ni), cobalt (Co), iron (Fe), aluminum (Al) and manganese (Mn), which is related to the cost of batteries. To better compare the characteristics of the state‐of‐the‐art cathodes that are being used in industry today (LiCoO_2_, LiNi_0.33_Co_0.33_Mn_0.33_O_2_, LiNi_0.80_Co_0.15_Al_0.05_O_2_, LiMn_2_O_4_, LiN_i0.5_Mn_1.5_O_4_, LiFePO_4_), Figure 1c–i provides the specific parameters of them.^[^ ^9^, ^10^, ^11^, ^12^, ^13^, ^14^, ^15^, ^16^ ^]^

LiCoO_2_ was successfully developed by Goodenough in the 1980s,^[^ ^17^ ^]^ which became the typical layered cathode material and remained the dominant cathode material for the portable electronics market. However, it can be seen that the content of Co is the lowest among above mentioned metals, leading to high cost. Additionally, toxicity and its adverse effects on the environment will be a major constraint on its development.^[^ ^18^ ^]^ Thus, Ni‐layered oxides mixed Co and Mn emerge as the time requires. LiNi_0.80_Co_0.15_Mn_0.05_ has an energy density of 760 kw kg^−1^ at 4.3 V thanks to the high nickel fraction.^[^ ^19^ ^]^ Nevertheless, A high nickel fraction would promote the cation disorder, resulting in the collapse of the crystal structure and the release of oxygen during the process of cycling.^[^ ^20^ ^]^ That is, although the cost of nickel is lower than that of Co, the low safety of ternary cathode materials mainly caused by the metal Ni cannot be ignored.^[^ ^21^, ^22^, ^23^ ^]^

Therefore, iron and manganese demonstrate great promise for cathode materials owing to their non‐toxicity, abundant resources, and low cost. And from a sustainability perspective, Mn‐based cathodes and LiFePO_4_ demonstrate an attractive prospect. Here it should be noted that the sustainability of materials is rated in terms of supply‐demand relations, mining technologies, environmental impacts, waste management, etc,^[^ ^24^ ^]^ and value 5 for sustainability in Figure 1c represents the highest level. LiFePO_4_ is commonly used in commercial electric vehicles owing to its high level of thermal stability and safety.^[^ ^25^ ^]^ However, the theoretical capacity of olivine LiFePO_4_ (170 mAh g^−1^) is significantly lower than that of other cathode materials (LiCoO_2_: 274 mAh g^−1^, LiNi_1−_ *_x_*_−_ *_y_* Co*_x_* Mn*_y_* O_2_: 273—285 mAh g^−1^). Moreover, compared with the theoretical density of LiCoO_2_, LiNiO_2_, and LiMn_2_O_4_ (5.1, 4.8, 4.2 g cm^−3^, respectively), LiFePO_4_ shows much lower theoretical density (3.6 g cm^−3^), which gives a seriously adverse effect on the energy density and specific capacity.^[^ ^26^ ^]^ Meanwhile, LiFePO_4_ has poor electronic conductivity and ionic conductivity, leading to initial capacity loss.^[^ ^27^ ^]^ Recently, Co‐free high‐voltage spinel LiN_i0.5_Mn_1.5_O_4_ has attracted attention due to its high operating voltage of 4.7 V (vs Li/Li^+^). However, high voltage brings about rapid capacity degradation and the decomposition of electrolytes.^[^ ^28^, ^29^, ^30^ ^]^ Hence, manganese‐based cathodes have lately received great attention for the development of Co‐free cathode materials.^[^ ^31^ ^]^ Compared with LiMn_2_O_4_ (148 mAh g^−1^), LiMnO_2_ possesses a higher theoretical capacity (285 mAh g^−1^).

As early as 2001, Ammundsen and Paulsen reviewed the progress of LiMnO_2_.^[^ ^32^ ^]^ In the past two decades, more researchers have extensively investigated LiMnO_2_ cathode, especially in terms of synthesis methods and modification manners. To get a more comprehensive understanding of LiMnO_2_ cathode, we deliver the review. This review is organized as follows. Firstly, in Section 2, the structural characteristics and drawbacks of LiMnO_2_ cathode are presented, as well as the synthesis methods discussed briefly. To improve the weaknesses of LiMnO_2_, modifications like element doping, surface coating, composites designing and finding compatible electrolyte are reviewed in Section 3. Finally, we describe our conclusions with an outlook for LiMnO_2_ cathode in Section 4.

## 2 LiMnO_2_

### 2.1 Crystal Structure and Comparison

**Figure 2:**
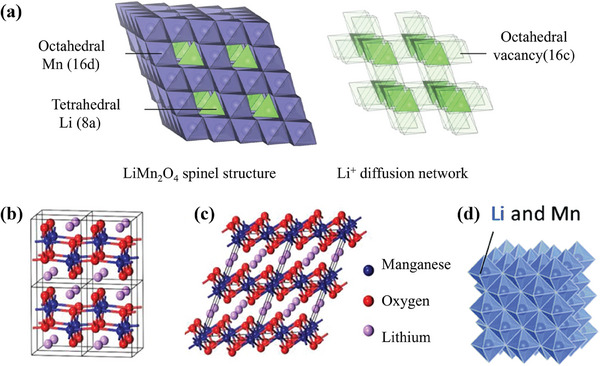
a) LiMn_2_O_4_ spinel crystal structure and Li^+^ ions diffuse rapidly between face‐sharing octahedral 16c and tetrahedral 8a sites. Reproduced with permission.^[^ ^26^ ^]^ Copyright 2020, Springer Nature. Crystal structure of b) o‐LiMnO_2_, c) m‐LiMnO_2_. Reproduced with permission.^[^ ^29^ ^]^ Copyright 2021, Elsevier. d) Metastable and cation‐disordered rocksalt‐type LiMnO_2_. Reproduced with permission.^[^ ^5^ ^]^ Copyright 2018, Royal Society of Chemistry

Layered LiMnO_2_ and spinel LiMn_2_O_4_ are two typical manganese‐based cathodes. The spinel LiMn_2_O_4_ belongs to the space group $Fd\bar{3}m$, where Li cations occupy the 8a site in 1/8 of the tetrahedron, Mn cations fill in the 16d site in 1/2 of the octahedral void, and O anions arranged by face‐centered cubes (FCC) lie in the 32e site (**Figure** **2**a).^[^ ^33^ ^]^ In this structure, unoccupied oxygen tetrahedra and oxygen octahedra are connected in a shared plane and line, which forms 3D interconnected channels for the diffusion of Li^+^.^[^ ^34^ ^]^ While layered LiMnO_2_ is a polymorphous compound including two quintessentially crystallographic structure (Figure 2b and c) and a cation disordered rocksalt polymorph (Figure 2d).^[^ ^5^, ^35^ ^]^ The orthorhombic LiMnO_2_ (referred to as o‐LiMnO_2_ in the following) having β‐NaMnO_2_‐like structures belongs to Pmnm space group (a = 0.2805 nm; b = 0.5757 nm; c = 0.4572 nm; Z = 2). In the crystal with a close‐packed oxygen lattice, all MnO_6_ octahedra shares a common edge with each other,^[^ ^36^ ^]^ as well as MnO_6_ and LiO_6_ are arranged in a corrugated interaction. The monoclinic LiMnO_2_ (herein referred to as m‐LiMnO_2_) is equipped with a structure of α‐NaFeO_2_ type, which belongs to the C2/m space group (a = 0.5439 nm; b = 0.2809 nm; c = 0.5395 nm; Z = 2), similar to LiCoO_2_ and LiNiO_2_.^[^ ^37^ ^]^ It can also be said that Li and Mn cations alternate to form zigzag layers along the (010) in o‐LiMnO_2_. Different from o‐LiMnO_2_, the Li and Mn cations of m‐LiMnO_2_ occupy octahedral site layers that are parallel to the (111) plane of the cubic oxygen sublattice.^[^ ^38^ ^]^ Consequently, Li cations fill in tetrahedral sites and Mn cations are in octahedral sites of the spinel LiMn_2_O_4_, while both Mn and Li cations occupy octahedral sites in the layered structure.^[^ ^39^ ^]^

Additionally, Yabuuchi et al. designed a cation‐disordered rocksalt‐type LiMnO_2_ by mechanical milling o‐LiMnO_2_. RIETAN‐FP (a software for crystal structure refinement) was used to analyze the structure of the samples before and after milling. The results show that o‐LiMnO_2_ is in a zigzag‐type layered structure with a high crystallinity. Whereas the sample lacks the structural features embodied in o‐LiMnO_2_ after mechanical milling. Meanwhile, the broad diffraction peaks of X‐ray diffraction (XRD) patterns suggest that the zigzag‐type structure of o‐LiMnO_2_ transforms into the cation‐disordered rocksalt phase.^[^ ^5^ ^]^

### 2.2 Challenges that LiMnO_2_ Faced

**Figure 3:**
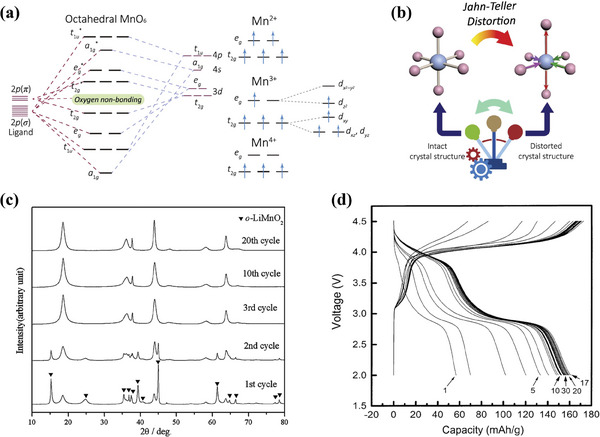
a) The molecular orbital energy diagram of the octahedral MnO_6_ and the electronic orbitals of Mn^2+^/Mn^3+^/Mn^4+^ ions. b) A schematic of the octahedral MnO_6_ before and after the J–T distortion. Reproduced with permission.^[^ ^43^ ^]^ Copyright 2021, Elsevier. c) XRD pattern evolution of the o‐LiMnO_2_ electrodes after different cycles. Reproduced with permission.^[^ ^45^ ^]^ Copyright 2007, Springer Nature. d) Charge and discharge characteristics of the o‐LiMnO_2_ for 30 cycles within the voltage range of 2–4.5 V. Reproduced with permission.^[^ ^48^ ^]^ Copyright 2004, Elsevier.

Unfortunately, commercial applications of layered LiMnO_2_ have been plagued by the following problems. The Mn cations in LiMnO_2_ all present as Mn^3+^.^[^ ^40^ ^]^ Three electrons in the d orbitals of high‐spin Mn^3+^ stay in the t_2g_ orbital with the same spin, and only one electron occupies an e_g_ orbital. It is important to note that e_g_ orbitals with $d_{x^{2-}}d_{y^{2}}\;$and $d_{z^{2}}$ are doubly degenerate orbitals obtained by splitting 3d orbitals (**Figure** **3**a). Consequently, this t_2g_^3^e_g_^1^ electronic configuration of Mn^3+^ results in the asymmetric occupation of e_g_ orbitals. Meanwhile, the electrons in the $d_{x^{2-}}d_{y^{2}}\;$and $d_{z^{2}}$ orbitals exhibit shielding for the Mn nucleus to varying degrees in different directions, resulting in an unstable state of central Mn^3+^. In order to maintain the stability of Mn^3+^, the two longitudinal Mn‐O bonds are gradually elongated, accompanying the shrinkage of the other four horizontal Mn‐O bonds (Figure 3b). In this case, the symmetry of MnO_6_ octahedron changes from O_h_ to D_4h_, giving rise to the Jahn–Teller (J–T) distortion.^[^ ^41^, ^42^, ^43^ ^]^ Simultaneously, the volume change from the cubic to tetragonal coordination results in the structural degradation of LiMnO_2_. Figure 3c illustrates the changes in XRD patterns of the o‐LiMnO_2_ electrodes after different cycles. The intensity of the o‐LiMnO_2_ peak gradually decreases with the progress of the cycle and some new peaks appear. After three cycles, there are no o‐LiMnO_2_ peaks in the XRD patterns, which provides compelling evidence that J–T distortion triggers the rapid structure transformation from layered into the spinel or the rock salt upon cycling.^[^ ^44^, ^45^ ^]^ It was confirmed by in situ X‐ray studies that o‐LiMnO_2_ was irreversibly converted to spinel structure.^[^ ^46^ ^]^ In addition, high stacking faults induced by structural disorder at Li/Mn sites are more likely to lead to phase transformation to a spinel‐like structure and display capacity fading in 3 V.^[^ ^47^ ^]^ According to the study by Lu et al.,^[^ ^48^ ^]^ additional plateau in the discharging curves (4.0 V) indicated spinel phase Li*_x_* Mn_2_O_4_ was formed from o‐LiMnO_2_ (Figure 3d). Based on charge/discharge curves analysis and structural studies, Molenda et al. proposed that phase transition (o‐LiMnO_2_↔LiMn_2_O_4_) was reversible in o‐LiMnO_2_.^[^ ^49^ ^]^ However, in‐depth examinations are required to understand the complex kinetics in the process of the phase transition.

On the other hand, the cycling performance of LiMnO_2_ is also restricted by the disproportionation of Mn element (Mn^3+^ → Mn^2+^ + Mn^4+^) during the discharge process. Seriously, Mn^2+^ is freely soluble in electrolyte solution. For one thing, Mn ions migrating and depositing on the anode will have major implications for solid electrolyte interphase (SEI). For another, Mn dissolution leads to the reduction of the active material, directly leading to structural degradation and capacity loss.^[^ ^50^, ^51^, ^52^ ^]^

It is concluded that phase transformation and manganese dissolution are the critical challenges for the application of layered LiMnO_2_. In addition, the loss of oxygen and the preparation of pure‐phase LiMnO_2_ also need to be cracked.

### 2.3 Synthesis and Electrochemical Performance

The synthetic route has a significant effect on the phase and structure of LiMnO_2_ as well as electrochemical properties. Similar to other cathode materials, the main synthesis methods of layered LiMnO_2_ include ion‐exchange method, solid‐state process, hydrothermal method, sol‐gel method and co‐precipitation.

In 1996, Armstrong et al. prepared the stoichiometric m‐LiMnO_2_ by the ion‐exchange method.^[^ ^53^ ^]^ First, NaMnO_2_ was prepared by heating Na_2_CO_3_ and Mn_2_O_3_ at high temperatures under the argon atmosphere and then refluxing the mixture of NaMnO_2_ and excess lithium compounds (LiCl or LiBr) in n‐hexanol. The initial charge capacity of obtained m‐LiMnO_2_ was up to 270 mAh g^−1^. In the same year, Delmas et al. also reported layered m‐LiMnO_2_ with metastable character by ion‐exchange process.^[^ ^54^ ^]^ It needs to be pointed out that refluxing experiments are carried out under the argon atmosphere and with a large excess of LiCl to protect the oxidation of any material. A differential scanning calorimetry (DSC) and a high temperature X‐ray diffractometer were applied to evaluate the thermal stability of m‐LiMnO_2_. Both measurement results indicated it is metastable. More specifically, exothermic effects at 300 °C are observed in the DSC curve. Meanwhile, the diffraction lines of o‐LiMnO_2_ disappear at 300 °C. In addition, Shao‐Horn et al. obtained a LiMnO_2_ material accompanied by spinel phase and orthorhombic LiMnO_2_ by ion exchange method.^[^ ^55^ ^]^

Zhou and co‐workers reported the one‐step synthesis of pure‐phase m‐LiMnO_2_ at a lower temperature (450 °C). Electrolytic manganese dioxide (EMD) was used as a manganese precursor to realize carbothermal reduction under the Ar atmosphere.^[^ ^56^ ^]^ An initial reversible capacity of 180 mAh g^−1^ is obtained in the synthesized sample. Using one‐step hydrothermal methods, the controlled preparation of LiMnO_2_ mixed orthorhombic and monoclinic phases was successfully achieved.^[^ ^57^ ^]^ It presented a higher discharge capacity (112.5 mAh g^−1^) after 50 cycles in a voltage range of 2.0–4.5 V than that of both structures (m‐LiMnO_2_: 94.5 mAh g^−1^, o‐LiMnO_2_: 106.8 mAh g^−1^). The fabrication of pure m‐LiMnO_2_ is considered to be relatively difficult because of its thermodynamic instability. Therefore, o‐LiMnO_2_ has been extensively investigated.^[^ ^58^, ^59^ ^]^

As early as the 1990s, there were studies through high‐temperature solid‐phase approaches to prepare layered LiMnO_2_, which was performed by calcining the mixture of MnO_2_ and lithium metal salts (LiOH/Li_2_CO_3_) at high temperatures (600–1000 °C) under an argon atmosphere.^[^ ^60^, ^61^ ^]^ To prevent manganese from being oxidized to the tetravalent state, a reducing agent like carbon was even required. Despite undergoing a phase transition to spinel in LiMnO_2_ during charging and discharging, an interesting phenomenon was found that a small amount of spinel in the original LiMnO_2_ cathode could improve electrochemical performance than pure LiMnO_2_ cathodes. In addition, its electrochemical performance was better than that of pure LiMnO_2_ cathodes. Lee et al. obtained the pure o‐LiMnO_2_ phase by a distinctive quenching process, which employed the reaction of LiOH with γ‐MnOOH at 1000 °C in an argon atmosphere.^[^ ^62^ ^]^ It delivered a high discharge capacity of 201 mAh g^−1^ in the first cycle and remained 200 mAh g^−1^ after 50 cycles at a current density of 0.4 mA cm^−2^ between 4.3 and 2.0 V. The group of Wang reported o‐LiMnO_2_ with a discharge specific capacity of 180—190 mAh g^−1^ by a two‐step solid‐state reaction.^[^ ^63^ ^]^ First, the precursors were prepared by firing mixtures of Mn_2_O_3_ and LiOH⋅H_2_O at 450 °C for 5 h, and then at higher 600 °C for 12 h with argon flow. Furthermore, one‐step synthesis by the solid‐state process using glucose as a reducing agent to prepare o‐LiMnO_2_ is also feasible.^[^ ^64^ ^]^ MnO_2_, LiOH⋅H_2_O and C_6_H_12_O_6_ mixed with a Li/Mn/C molar ratio of 5/4/2 were well ground, and then annealed at 750 °C for 15 hours in a tube furnace under nitrogen flow. In this way, heating temperature and time need to be strictly controlled, otherwise impurity phases are accompanied. However, severe particle agglomeration and high energy consumption are inevitable problems in solid‐phase synthesis.

The hydrothermal method with low energy consumption is an ideal way to prepare o‐LiMnO_2_ for getting uniform and fine particles. In the processes of two‐step hydrothermal synthesis of LiMnO_2_, there are three precursors (γ‐MnOOH, Mn_3_O_4_, and Mn_2_O_3_) formed by the redox reaction of the manganese source. The crystalline o‐LiMnO_2_ can be obtained by reacting γ‐MnOOH as the precursor with LiOH solution in a Teflon‐lined autoclave at 180 °C for 24 h.^[^ ^65^ ^]^ The γ‐MnOOH nanorods as precursors were synthesized by magnetically stirring a mixed solution of polyethylene glycol (PEG‐10000) and Mn(NO)_3_ at 140 °C for 24 h, namely a novel polymer‐assisted low‐temperature hydrothermal method. The attempts to synthesize o‐LiMnO_2_ hydrothermally with Mn_3_O_4_ as a precursor were completed by Komaba et al. in 2002.^[^ ^66^, ^67^ ^]^ Dark brown Mn_3_O_4_ powders were prepared by stirring the aqueous mixed solution of Mn(CH_3_COO)_2_ and KOH at 80 °C for 24 h with bubbling O_2_ gas. It is important to wash the Mn_3_O_4_ powders with deionized water until the pH value of 7 for the synthesis of o‐LiMnO_2_. Interestingly, replacing γ‐ MnOOH and Mn_2_O_3_ with Mn_3_O_4_ as a precursor can obtain highly crystalline o‐LiMnO in hydrothermal synthesis, enabling initial reversible capacity of 210 mAh g^−1^.^[^ ^68^ ^]^ Nanocrystalline Mn_3_O_4_ particles can also be prepared through the reduction of high‐valent manganese compounds (KMnO_4_) with methanol or ethanol in an autoclave at the temperature of 80—100 °C for 12–48 h.^[^ ^69^ ^]^ In addition, Yang group introduced a rapid method to synthesize nanosized o‐LiMnO_2_, which was based on microwave‐solvothermal approach. This technique mainly uses α‐Mn_2_O_3_ as the precursor mixed with LiOH·H_2_O for ≈30 min at a low temperature of 160 °C.^[^ ^70^ ^]^ The microwave hydrothermal synthesis was carried out in the microwave digestion system. Introducing hydrazine hydrate (N_2_H_4_⋅H_2_O) as a reducing agent in the hydrothermal route, submicron‐sized o‐LiMnO_2_ crystals can be successfully prepared by using LiOH and the precursors of porous Mn_2_O_3_ at the temperature of 180 °C for 16 h.^[^ ^71^ ^]^ The Mn_2_O_3_ precursor was formed by calcining MnCO_3_ under the air atmosphere at 620 °C for 6 h. The submicron‐sized o‐LiMnO_2_ displayed a superior discharge capacity of 216 mAh g^−1^.

**Figure 4:**
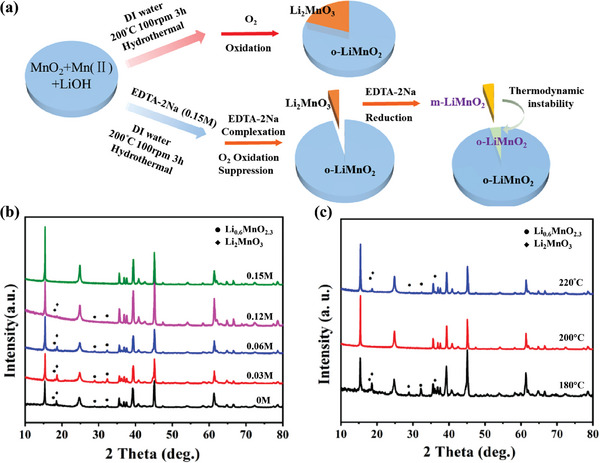
a) The supposed mechanism of the function of EDTA‐2Na in the hydrothermal reaction system to eliminate the Li_2_MnO_3_ and form pure o‐LiMnO_2_. XRD patterns of the resulting LiMnO_2_ samples at b) different EDTA‐2Na concentrations at 200 °C and c) different hydrothermal temperatures at 0.15 M EDTA‐2Na. Reproduced with permission.^[^ ^79^ ^]^ Copyright 2020, Elsevier.

Nevertheless, the time‐consuming and complex procedures involved in multi‐step processes are the undeniable disadvantages of the two‐step synthesis mentioned above. Hence, the facile and simple one‐step hydrothermal approach was chosen to obtain LiMnO_2_ materials.^[^ ^72^, ^73^ ^]^ This way is conventionally achieved by employing the reactants of Mn source (Mn_2_O_3_/MnO_2_) and LiOH·H_2_O aqueous solution in a Teflon‐lined autoclave. Unfortunately, this method may introduce small amounts of impurities (e.g., Li_2_MnO_3_) in samples.^[^ ^74^ ^]^ Li_2_MnO_3_ is also a layered lithium‐manganese oxide with an α‐NaFeO_2_ type structure and the C2∖m monoclinic crystal system. In this structure, Li cations occupy the interslab octahedral position in the rock salt structure, and 1/3 of Li cations and 2/3 of Mn cations occupy slab octahedral sites of the transition metal layer,^[^ ^75^ ^]^ so it can also be written as the conventional layered structural formula of Li[Li_1/3_Mn_2/3_]O_2_. There are two hypotheses regarding the explanation of the electrochemical activity of Li_2_MnO_3_. With the aid of flame emission, atomic absorption, X‐ray photoelectron spectroscopy (XPS), thermogravimetric analysis coupled with mass spectrometry (TGA/MS), Bruce et al. investigated the electrochemical activity of Li_2_MnO_3_ in organic electrolytes. They indicated that the activity of Li_2_MnO_3_ could be attributed to the exchange of Li^+^ by H^+^ in organic electrolytes rather than removal of O^2−^, and there was no oxidation of Mn^4+^ to Mn^5+^.^[^ ^76^ ^]^ Another hypothesis was proposed by Dahn in 2002. They believed oxygen was irreversibly released during the first charge to 4.8 V in Li/Li[Ni*_x_* Li_(1/3‐2_ *_x_*_/3)_Mn_(2/3‐_ *_x_*_/3)_]O_2_ material.^[^ ^77^ ^]^ In 2006, Armstrong et al. used in situ differential electrochemical mass spectrometry (DEMS) to directly detect the oxidation of O^2−^ in Li_2_MnO_3_ structure and the accompanying Li^+^ extraction, which provided a reliable basis for the mechanism of oxygen loss to the electrochemical activity of Li_2_MnO_3_.^[^ ^78^ ^]^ Ethylenediaminetetraacetic acid disodium salt (EDTA‐2Na) acted as both a chelating reagent and reducing agent to prepare pure o‐LiMnO_2_ via hydrothermal synthesis. EDTA‐2Na not only prevents residual oxygen from oxidation in the reacting system effectively but also ingeniously suppresses the formation of Li_2_MnO_3_ (**Figure** **4**a).^[^ ^79^ ^]^ Figure 4b,c show the influence of the concentration of EDTA‐2Na and the reactive temperatures on the appearance of Li_2_MnO_3_, respectively_._

Particularly, the conditions for soft hydrothermal synthesis can affect the properties of the material. Thus, adjusting the synthesis conditions is an effective way to improve electrochemical performance. A clear understanding of the formation and growth mechanism of materials (including the nucleation time and growth process) is usually expected when conducting synthetic experiments, and in situ hydrothermal synthesis might be a good way to do this.^[^ ^80^ ^]^

**Figure 5:**
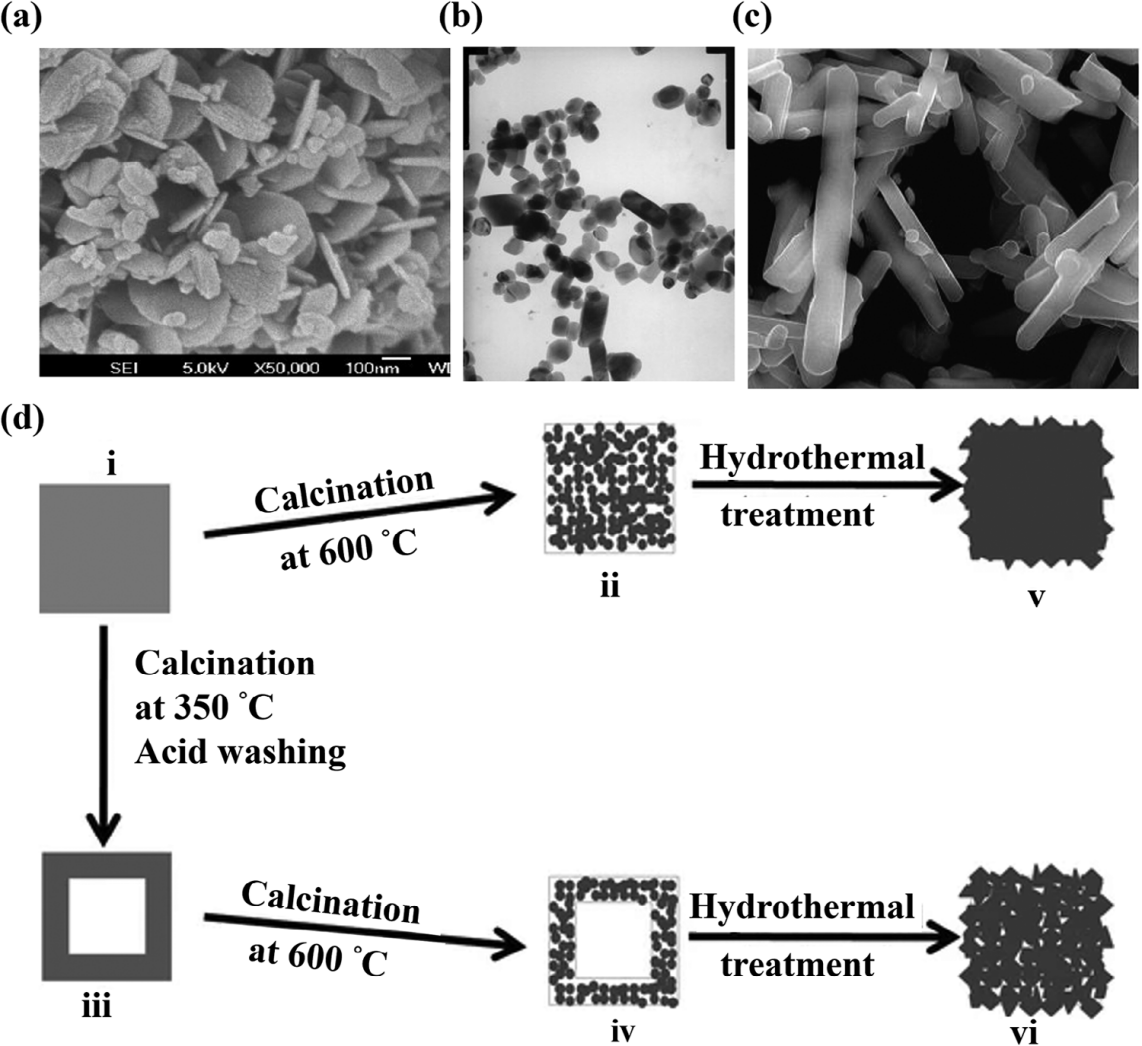
a) SEM images of o‐LiMnO_2_ nanoplates. Reproduced with permission.^[^ ^81^ ^]^ Copyright 2009, Elsevier. b) TEM images of LiMnO_2_ powders. Reproduced with permission.^[^ ^82^ ^]^ Copyright 2010, Elsevier. c) SEM images of the LiMnO_2_ nanorods. Reproduced with permission.^[^ ^83^ ^]^ Copyright 2016, Elsevier. d) Schematic illustration of the preparation of the final LiMnO_2_ microcubes (v and vi). Reproduced with permission.^[^ ^84^ ^]^ Copyright 2013, Springer.

**Figure 6:**
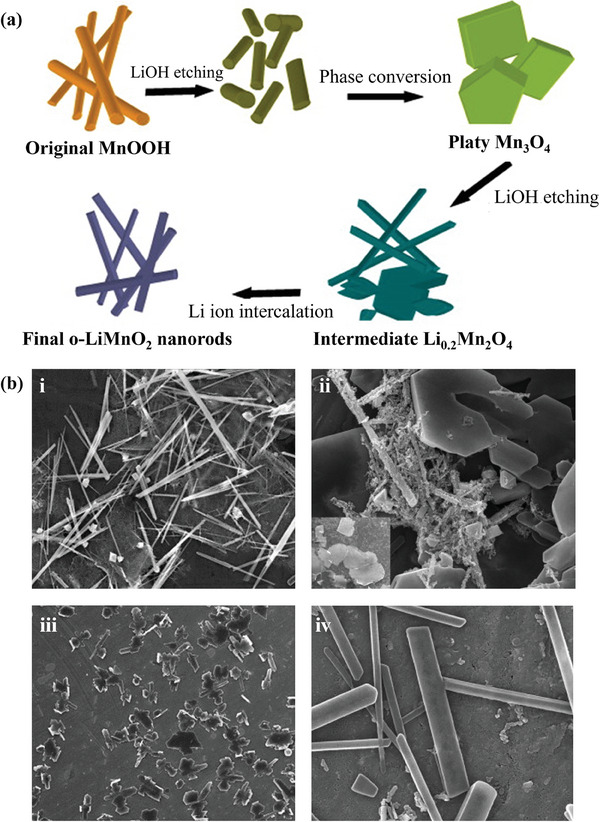
a) A scheme showing the formation of o‐LiMnO_2_ nanorod. b) SEM micrograph of sample powders with different hydrothermal times. i) the MnOOH precursor, ii) 2 h incorporation, iii) 3 h incorporation, and iv) 4 h incorporation). Reproduced with permission.^[^ ^85^ ^]^ Copyright 2007, Elsevier.

To the best of our knowledge, the electrochemical performance is heavily affected by the morphology and structure of the material. Accordingly, LiMnO_2_ with specific nanostructures and morphologies (e.g., nanoplates, nanoparticles, nanorods, and microcubes) have spurred an intensive search for excellent electrochemical performance (**Figure** **5**).^[^ ^81^, ^82^, ^83^, ^84^ ^]^ In 2007, Liu et al. used needle‐like MnOOH as the precursor to prepare o‐LiMnO_2_ with a nanorod‐like shape. Notably, the platy shape, platy with irregular shape and rod‐like crystals corresponded to the appearance of Mn_3_O_4_ phase, cubic Li_0.2_Mn_2_O_4_ phase, and pure o‐LiMnO_2_ after 2 h, 3 and 4 h hydrothermal treatment, respectively (**Figure** **6**).^[^ ^85^ ^]^ It is also a good evidence that LiOH has the two important roles in providing a source of lithium and controlling both morphology and particle size. Xiao et al. prepared orthorhombic LiMnO_2_ nanoparticles and LiMnO_2_ nanorods by different hydrothermal methods.^[^ ^86^ ^]^ Compared with LiMnO_2_ nanoparticles prepared through simple one‐step routes, LiMnO_2_ nanorods synthesized by γ‐MnOOH precursors showed a higher discharge capacity (200 mAh g^−1^) as well as better cyclability (180 mAh g^−1^ after 30 cycles) due to favorable electronic transport of 1D electronic pathways. Zhao et al. combined Mn_2_O_3_ nanorods as a template and thermal decomposition process to prepare o‐LiMnO_2_, which delivered outstanding performance. As a consequence, the one‐dimensional crystalline nanostructure could effectively promote the transport of charge/electron and increase the electrode‐filled ratio.^[^ ^83^ ^]^

To overcome the difficulty in synthesis of layered LiMnO_2_, there are some novel methods developed, including the emulsion‐drying method, reverse‐microemulsion preparation, microwave irradiation, in.situ oxidation coupling with ion exchange, and in situ carbothermal reduction. It is reported that o‐LiMnO_2_ materials by emulsion‐drying method show only a 10% capacity loss after 300 cycles at 45 mA g^−1^. The emulsion‐drying method could mix cations homogeneously at the atomic level and obtain fine single‐crystal oxide cathode materials.^[^ ^87^ ^]^ Reverse‐microemulsion preparation is another nice way to allow cations to mix and interact in an atomic scale. Obtaining a thermodynamically stable and transparent microemulsion, in which nano‐sized water is well dispersed into the oil phase, is the key to reverse microemulsion preparation.^[^ ^48^ ^]^ One of the new technologies based on hydrothermal synthesis is microwave hydrothermal. With the advantage of microwave radiation, it significantly reduces reaction time and temperature in some processes.^[^ ^88^ ^]^ Li et al. described the synthesis of high‐purity and highly crystallized o‐LiMnO_2_ via the process of in situ oxidation coupling with ion exchange.^[^ ^89^ ^]^ During the synthesis process, MnOOH was produced by the oxidation of Mn(OH)_2_ with the oxidizing agent (NH_4_)_2_S_2_O_8_ in a LiOH‐existing strong basic environment. Despite 80 °C, deoxygenated distilled water and nitrogen were required to eliminate the impact of oxygen in the air. Komarnenib's group prepared nanorod‐like o‐LiMnO_2_ with high purity and excellent electrochemical behavior by in situ carbothermal reduction.^[^ ^90^ ^]^ In this synthesis process, MnO_2_, LiOH·H_2_O and affordable glucose (C_6_H_12_O_6_) as a reducing agent were homogeneously mixed followed by high temperature treatment in an argon atmosphere for several hours. Additionally, sol‐gel and co‐precipitation methods have also been used for the synthesis of LiMnO_2_. As for the sol‐gel approach to get LiMnO_2_, citric acid is a common chelating agent.^[^ ^91^ ^]^ Zeng group obtained spherical o‐LiMnO_2_ powders with a discharge capacity of 152 mAh g^−1^ by a carbonate coprecipitation method using NH_4_HCO_3_ and MnCl_2_ as raw materials.^[^ ^92^ ^]^

Briefly, it is relatively difficult to synthesize pure layered LiMnO_2_ for its thermodynamic sub‐stability, especially for monoclinic LiMnO_2_. Li_2_MnO_3_ and manganese oxide as impurities are frequently seen in the XRD analysis of the product. So, to avoid the various side reactions during synthesis, the use of inert gas is the recommended choice. Both solid‐state synthesis and hydrothermal synthesis should strictly control the Li/Mn ratio, reaction temperature, and reaction time. Moreover, in situ detection techniques can be used for precise material preparation.

## 3 Modifications

To overcome the drawbacks mentioned in Section 2, tremendous efforts such as element doping, surface coating, nanocomposite designing and compatible electrolyte have been adopted to improve the electrochemical performance of LiMnO_2_ cathode materials.

### 3.1 Element Doping

Element doping is one of the most extensive approaches for modifying cathode materials to improve the basic physical properties of materials. The elements doping can suppress phase transitions, and enhance capacity. For LiMnO_2_ cathode, the research on doping strategies mainly includes element types, element content, doping sites, synthesis conditions, morphology, and structural effects. The efficacy of element substitution by doping is mainly manifested in the following aspects: 1) inhibiting J–T distortion and making structures stable for improved cycling performance; 2) providing favorable Li^+^ diffusion. For substituting at the Mn site, doping ions with smaller radius (Al^3+^, Co^3+^, Cr^3+^) than Mn^3+^ result in a contracted crystal lattice and microstrains owing to the shorter and stronger transition metal‐oxygen bonds than Mn─O bonds; substitutes of larger radius (Y^3+^) show larger lattice parameters and lead to the free movement of Li^+^ in the oxide, which makes it possible to increase the rate of capability. For substituting at the Li site, the metal element with a larger radius ion can provide a pillaring effect to enhance the stability of cathode materials. For instance, Li_0.53_Na_0.03_MnO_2_ with a pseudo‐spinel structure displays an increasing discharge capacity, which reaches 200 mAh g^−1^ in the 50th cycle.^[^ ^93^ ^]^ Up to now, various doping elements have been found to improve the electrochemical properties of layered LiMnO_2_, including Na, Cu, Mg, Zn, Fe, Ni, Al, Co, Cr, Y, In, S, Ti, V, Nb, Ru, B, F, BO_3_^3−^, PO_4_^3−^and SiO_3_^3−^.

#### 3.1.1 Cations Doping

##### Divalent Cations

Transition metal elements usually occupy the Mn site of lithium manganese oxide to improve electrochemical performance. The ionic radius of the doping Cu^2+^ (0.072 nm) is larger than that of Mn^3+^, which is beneficial for faster mobility of Li^+^ in bulk, contributing to the decreased charge transfer impedance and Warburg impedance of LiMnO_2_. Such lower resistance has an important role in improving high‐rate capability by 65.5%.^[^ ^94^ ^]^ Unlike the doping of transition metal elements, the Mg may occupy both Li and Mn sites. During the charge/discharge cycling, the presence of Mg at the Li sites is beneficial for maintaining the stability of the structure but reduces the electrochemical extraction of lithium ions.^[^ ^95^ ^]^

Suresh et al. investigated the doping effect of Zn and Fe elements using the ion‐exchange method.^[^ ^96^ ^]^ It was only 5% Zn/Fe (LiMn_0.95_Zn_0.05_O_2_/LiMn_0.95_Fe_0.05_O_2_) substituted into LiMnO_2_ that could exhibit a better discharge capacity of 180 and 193 mAh g^−1^, respectively. The substitution of Zn can improve the retention of capacity. However, increasing the content of Zn leads to the capacity fading due to decreasing of electrochemically active Mn^3+^. The decreased intensity of Mn^3+^/Mn^4+^ redox peak in the cyclic voltammograms (CV) curves confirms it.

##### Mixed Bivalent and Trivalent Cations

Quine et al. reported that Li*_x_* Mn_0.95_Ni_0.05_O_2_ with the O3 (α‐NaFeO_2_) structure formed via an ion‐exchange route. Li*_x_* Mn_0.95_Ni_0.05_O_2_ delivered a high capacity of 220 mAh g^−1^ above 2.5 V.^[^ ^97^ ^]^ Inevitably, a phase transition to spinel occurred after the cycling. In that case, Ni substituted in the layered LiMnO_2_ was in a state of Ni^2+^ thus accompanied by a reaction from Mn^3+^ to Mn^4+^. Drawing support from the x‐ray absorption spectroscopy (XAS) spectrum, Wadati et al. pointed out that Li deficiencies could not be created by the Ni^2+^ valence and the change from Mn^3+^ to Mn^4+^.^[^ ^98^ ^]^ However, the Ni^3+^ was observed in the CV curves, corresponding to anodic peak of 3.05 V.^[^ ^95^ ^]^ In addition, when 5% Ni is substituted, the compound provides the highest capacity of 220 mAh g^−1^ but is followed by a rapid capacity decay on repeated cycling. The high Ni contents can provide stable capacity, due to the decrease of Mn^3+^ contents, while the loss of capacity is inevitable. Nahm and co‐workers found that O2‐Li_0.7_[Ni_1/6_Mn_5/6_]O_2_ instead of Li_0.7_[Li_1/6_Mn_5/6_]O_2_ exhibited no phase change after 30 cycles.^[^ ^99^ ^]^ In the first cycle, this sample delivered a discharge capacity of 198 mAh g^−1^ between the voltage of 4.6 and 2.0 V. Meanwhile, it exhibited a 96% capacity retention after 25 cycles at 1/3 C. Therefore, Ni doping can exert a positive effect on the stability of the structure and the retention of capacity.

##### Trivalent Cations

In the 1990s and early 2000s, interests in modifying layered LiMnO_2_ were focused more on the solid solution formed between LiMnO_2_ and LiMO_2_ analogues (LiAlO_2_, LiCoO_2_, and LiCrO_2_). Although pure LiAlO_2_ is electrochemically inert, the solid solution consisting of LiAlO_2_ and other lithiated transition‐metal oxides probably possesses a high intercalation potential, leading to high energy density.^[^ ^100^ ^]^ And two effects can explain why Al^3+^ doping can stabilize the monoclinic phase:1) AlO_6_ octahedra is not distorted because of non‐J‐T Al^3+^; 2) the smaller size of Al^3+^ (0.054 nm) has advantages in building stable transition metal‐oxygen bond.^[^ ^101^, ^102^ ^]^ Chiang et al. indicated that the Al‐doped LiMnO_2_ contained the monoclinic and orthorhombic ordered rock‐salt structures, and exhibited better cycling performance and higher discharge capacities under the test conditions of elevated temperatures.^[^ ^103^ ^]^ But m‐Li*_x_* Al_0.05_Mn_0.95_O_2_ showed a higher discharge capacity of 188 mAh g^−1^ as well as higher energy densities of 602 Wh kg^−1^ than o‐Li*_x_* Al_0.05_Mn_0.95_O_2_ (146 mAh g^−1^ and 465 Wh kg^−1^) at 55 °C. The doping of an element not only directly improves the electrochemical performance of materials, but also affects the structure and morphology of materials. Regan et al. elucidated the impacts of Al doping on the structures and surface properties of material^[^ ^104^ ^]^ The existence of Al favors on Mn‐Li inversion in alternating slabs of the LiAl*_x_* Mn_1‐_ *_x_* O_2_. Moreover, it is manifested by XPS that Al is uniformly incorporated in the whole grains up to the limit of its solubility. And no aggregation of Al is observed on the surface. The group of Yang described the facile hydrothermal synthesis of o‐LiMn_1‐_ *_x_* Al*_x_* O_2_ and investigated the changes in the morphology with varying Al content in the layer.^[^ ^105^ ^]^ The results indicate that Al^3+^ in a small content contributes to growth in the b‐axis direction (i.e., (010) direction) and formation of cuboid crystals, but the growth is inhibited and changes to cubic crystals with a high content of Al^3+^. In 1999, Robertson prepared Co‐substituted Li(Mn_1‐_ *_y_* Co*_y_*)O_2_ in the range 0≤*y*≤0.5 using a solution‐based route with the assistance of ion exchange.^[^ ^106^ ^]^ On the one hand, it pointed out that Co entered the layered LiMnO_2_ as Co^3+^ rather than Co^2+^. On the other hand, it indicated that higher content of Co caused somewhat lower capacities, and the best amount of doping was 2.5%.^[^ ^107^, ^108^ ^]^ The LiMn_1‐_ *_y_* Co*_y_* O_2_ from the hydrothermal synthesis gave different optimal doping content (10%).^[^ ^109^ ^]^ Doping of Al, Co, and Cr makes sense in stabilizing LiMnO_2_ structure and improving cycle retention. In addition, all doped compounds show contracted crystal lattices by comparison with nonsubstituted LiMnO_2_ due to a relatively small radius of doping cations. Although LiMn_1‐y_Co_y_O_2_ and LiMn_1‐y_Al_y_O_2_ undergo a spinel phase transition at 4 V,^[^ ^110^, ^111^ ^]^ this has almost no harmful effect on cycling performance and even increases discharge capacity.^[^ ^112^ ^]^ But low levels of Cr^3+^‐substituted materials show insignificant transformation in cycling.^[^ ^113^ ^]^ The key points of Cr doping depend on 1) forming a continuous solid solution in LiMnO_2_ across the whole range of composition from LiMnO_2_ to LiCrO_2_;^[^ ^114^ ^]^ 2) more regular local coordination symmetry around Cr^3+^ than Mn^3+^; 3) higher coordination between Cr^3+^ and oxygen than Mn^3+^.^[^ ^115^ ^]^ Furthermore, with the help of ^6^Li magic‐angle spinning NMR spectroscopy, it has been found that Cr^3+^ doping can partially disrupt the Mn‐Mn antiferromagnetic correlations, which is important for the stabilization of the layered structure over the orthorhombic structure.^[^ ^114^, ^116^ ^]^ Alternatively, Pang observed that even though Cr doping into o‐LiMnO_2_ brought a change in the topography from orthorhombic to monoclinic geometry employing Pechini's synthesis method and XRD patterns,^[^ ^117^ ^]^ it does not matter on improving cycling performance and reversible capacity. Xiao et al. reported the rheological phase method to prepare m‐LiMn_1‐_ *_x_* Cr*_x_* O_2_,^[^ ^118^ ^]^ forming ultrafine spherical particles with the size of 60–200 nm. Relative to the preparation through solid‐state reaction at the higher temperature of 1000 °C, LiMn_0.85_Cr_0.15_O_2_ by rheological phase method calcined at 800 °C yields a much higher initial discharge capacity (180 mAh g^−1^) and capacity retention as well (94% after 40 cycles).

Y^3+^ as a substituent in place of Mn^3+^ in LiMnO_2_ was first explored by Zong and partners.^[^ ^119^ ^]^ There is no 4 V plateau associated with spinel LiMn_2_O_4_ in the CV and charge/discharge curves of the Li/LiMn_0.98_Y_0.02_O_2_ cell, which can be attributed to the pillaring effect of Y^3+^. In addition, doping with a low content of Fe is beneficial for capacity and cycling stability.^[^ ^96^ ^]^ Whereas, Myung found o‐LiMnO_2_ of Fe substitution by a hydrothermal reaction obtained much lower capacity than that of undoped one,^[^ ^120^ ^]^ which is down to quasi‐reversible Fe^4+^/Fe^3+^ redox couple and gives unimpressive battery performance. The introduction of Ti^3+^ into o‐LiMnO_2_ is an effective attempt to moderate the phase evolution and stabilize the structure.^[^ ^121^ ^]^ Unfortunately, the secondary phase m‐Li_2_MnO_3_ in LiMnO_2_ has a low conductivity. By doping Ti^3+^ into LiMnO_2_, c‐LiTiO_2_ with better electrical conductivity (ca. 10^−6^ S cm^−1^) can replace above mentioned secondary phase. And Ti─O bonds are stronger than Mn‐O bonds, which militates in favor of improved structural stability of o‐LiMn_1‐_ *_x_* Ti*_x_* O_2_. In addition, B‐doped m‐LiMnO_2_ was prepared by carbothermal reduction using LiOH and MnO_2_ as reactants under the argon atmosphere.^[^ ^56^ ^]^ Even though the transformation from layer to spinel cannot be suppressed by B‐doping, the cycle performance and Coulombic efficiency are improved. 5% B‐doping (m‐LiB_0.05_Mn_0_._95_O_2_) gives the best electrochemical performance among B‐doped samples with different ratios, particularly at elevated temperatures (60 °C). The average Coulombic efficiency increases from 69.7% to 99.1% at 60 °C.

#### 3.1.2 Anions Doping

Aside from cations doping, doping anions is another feasible approach to modify the LiMnO_2_ cathode. The S‐doped LiMnO_2_ can deliver a discharge capacity of 220 mAh g^−1^ after 50 cycles with a voltage of 2.0–4.6 V.^[^ ^122^ ^]^ The F substitution can stabilize the host structure due to high electronegativity, showing better cycle performance compared with F‐free LiMnO_2_.^[^ ^123^ ^]^ Because the S^2−^ (0.184 nm) and F^−^ (0.136 nm) are greater than the O^2−^ (0.132 nm), the lattice constant of LiMnO_2_ doped with S^2^ and F^−^ increases. However, above mentioned S and F‐doping cannot ultimately suppress the transformation from layer to spinel.

Larger polyanions (BO_3_^3−^, PO_4_^3−^and SiO_3_^3−^) in place of O^2−^ ions in nonstoichiometric Li*_x_* MnO_2_ can generate an open 3D framework, which enhances the mobility of Li^+^ ions. Decreased Warburg impedance is also observed, which is reflected in the improved electrochemical properties of the high‐rate charge/discharge capability. Unfortunately, polyanion doping presents increased charge‐transfer resistance due to the poor electrochemical activity of polyanions relative to cations. Surprisingly, although the discharge capacity of Li*_x_* MnO_1.99_X_0.01_ (X = BO_3_^3−^, PO_4_^3−^and SiO_3_^3−^) has decreased, the cycling ability has been improved.^[^ ^94^ ^]^

#### 3.1.3 Co‐Doping

Furthermore, doping multiple elements into LiMnO_2_ endows electrodes with a synergistic effect. Suresh doped 5% Ni and 5% Fe into LiMnO_2_,^[^ ^124^ ^]^ forming a pure and homogeneous phase with a layered structure. The low content of Ni and F exhibited a discharge capacity of 250 mAh g^−1^ at the rate of 0.1 C, and improved the cycling stability. The co‐doping of cations and anions, such as Li^+^ and F^−^ ions, into o‐LiMnO_2_ has been realized through a solid‐state reaction.^[^ ^125^ ^]^ Scanning electron microscopy characterization indicates o‐Li_1.07_Mn_0.93_O_1.92_F_0.08_ presents a smooth surface even after cycling. Co‐doping of Li^+^ and F^−^ ions reduces the contact area with the electrolyte as well as suppresses the reaction of the Mn dissolution. Eventually, the products improved cycling stability and rate capability at a higher temperature (55 °C). However, doped LiMnO_2_ with multiple elements still shows some reserved disadvantages, such as low specific capacity.

Su investigated In^3+^ and S^2−^ co‐doped o‐LiMnO_2_ synthesized via the hydrothermal method.^[^ ^126^ ^]^ Compared to pristine and single doping (In or S), dual doping reduced the crystallinity of LiMnO_2_. Meanwhile, In and S dual doping exhibited the partial structural transformation from orthorhombic to spinel after 10 cycles at 50 mA g^−1^, but excellent cycle performance was obtained at various discharge current densities. In brief, In and S dual doping provides a great method to improve the rate performance of LiMnO_2_ cathode.

#### 3.1.4 Theoretical Calculations on Doping Effect

As a quantum mechanical method for the study of the electronic structure of multi‐electron systems, density functional theory (DFT) is very effective in offering a deeper elaboration of elemental doping. An ab initio study pointed out that 10% Co‐substitution makes the antiferromagnetic spin orthorhombic structure more stable than the monoclinic structure, and 60% substitution helps stabilize the disordered local moment (DLM) rhombohedral layered structure over the ferromagnetic orthorhombic phase and offers easy migration of lithium during intercalation.^[^ ^127^ ^]^

Prasad et al. reported their first‐principles calculations of doped rhombohedral LiMnO_2_ against J–T distortion.^[^ ^128^ ^]^ All calculations indicate that, for dopants, their oxidation state is the first significant factor for effectively stabilizing the rhombohedral structure, and then for a certain oxidation state, the t_2g_ or e_g_ subshell filling is the second. In addition, results show that divalent dopants (such as Mg, Zn) are more effective in the suppression of the J–T distortion than trivalent dopants because each divalent dopant can remove two Mn^3+^ ions from the sublattice.^[^ ^129^ ^]^ Based on generalized gradient approximation, it is found that divalent dopants (Mg and Co) destabilized the monoclinic structure than the trivalent dopant (Fe).^[^ ^130^ ^]^ Using the hybrid eigenvector‐following and DFT approach, Grey groups studied the effect of trivalent dopants on the Mn migration leading to spinel transformation, and found that dopants with a small ionic radius (Al^3+^ and Cr^3+^) can raise the migration barrier of Mn, but only Cr^3+^ does not move to tetrahedral sites within the Li layer.^[^ ^131^ ^]^ Kong et al. investigated the effects of ten cationic (Mg, Ti, V, Nb, Fe, Ru, Co, Ni, Cu, and Al) and two anionic (N and F) dopants of LiMnO_2_ using ab initio DFT simulations.^[^ ^132^ ^]^ The findings indicate that Mg, Ti, V, Nb, Ru as well as F can effectively reduce the redox potential, Ti, V, Nb, and Ru can increase the hole conductivity to inhibit the formation of the hole polaron. However, only Ni can decrease the diffusion batteries of Li^+^ by 0.23 V and only the N‐doped phase is thermodynamically unstable. When doping at the Li site, it shows unfavorable thermodynamics than Mn‐site doped configurations.^[^ ^133^ ^]^ Similarly, Khang Hoang indicates that substitutes (Al, Fe) are more favorable at the Mn site.^[^ ^134^ ^]^

To summarize, a systematical comparison of ionic radius and electrochemical performance of different elements doped into LiMnO_2_ is presented in **Table** **1**. Element doping is effective in stabilizing structures and improving the electrochemical performance of LiMnO_2_. To one degree or another, it ameliorates the adverse effects of transformation from a layer to a spinel crystal structure. For Co and Cr doping, it shows good capacity retention upon cycling. However, element doping in LiMnO_2_ still suffers from some limitations. For instance, it is inevitable for Al‐doped LiMnO_2_ to convert into a spinel phase at 4 V. Keeping the high capacity retention for long cycles is still the biggest problem. Decreasing the amount of Li also needs to be solved during the process of electrochemical extraction, when a dopant occurs at the Li site

**Table 1:** Comparison of ionic radius and electrochemical performance of different elements doped into LiMnO_2_.

Doping ions	Ionic radius [nm]	Cathode	Voltage range [V vs Li/Li^+^]	Initial discharge capacity [mAh g^−1^]/current density	Discharge capacity [mAh g^−1^]/retention/cycles	Reference
Na^+^	0.102	Li_0.53_Na_0.03_MnO_2_	2.4–4.5	180/0.2 C	200/111.1%/50	[93]
Cu^2+^	0.072	Li_0.75_Mn_0.99_Cu_0.012_O_2_	2.0–4.3	219/60 mA g^−1^	≈195/89%/50	[94]
Mg^2+^	0.072	LiMn_0.95_Mg_0.05_O_2_	2.0–4.5	150/1/7 C	≈150/100%/30	[95]
Zn^2+^	0.074	LiMn_0.95_Zn_0.05_O_2_	2.0–4.5	185/–	≈178/96.2%/30	[96]
Ni^2+^/Ni^3+^	0.069/0.056	LiMn_0.95_Ni_0.05_O_2_	2.4–4.8	220/25 mA g^−1^	≈215/97.7%/50	[97]
		LiMn_0.95_Ni_0.05_O_2_	2.0–4.5	220/1/7 C	≈180/81.8%/30	[95]
		Li_0.7_[Ni_1/6_Mn_5/6_]O_2_	2.0–4.8	198/1/3 C	190/96%/.25	[99]
Al^3+^	0.054	m‐Li*_x_* Al_0.05_Mn_0.95_O_2_ o‐Li*_x_* Al_0.05_Mn_0.95_O_2_	2.0–4.4 2.0–4.4	≈150/26.1 mA g^−1^ ≈25/75 mA g^−1^	188/125.3%/50 146/636.6%/100	[103] [103]
		Li_0.93_Mn_0.96_Al_0.04_O_2_	2.0–4.3	175/0.1 C	145/82.9%/25	[105]
Co^3+^	0.055	Li_0.9_Mn_0.9_Co_0.1_O_2_	2.6–4.8	210/0.1 mA cm^−2^	162/77.1%/50	[97]
		Li*_x_* Mn_0.975_Co_0.025_O_2_	2.4–4.6	200/25 mA g^−1^	188/94%/100	[107]
		LiMn_0.9_Co_0.1_O_2_	2.0–4.3	71/45 mA g^−1^	170/239.1%/100	[109]
Cr^3+^	0.061	LiMn_0.9_Cr_0.1_O_2_	2.5–4.5	200/0.1 C	200/100%/30	[117]
		R‐LiMn_0.85_Cr_0.15_O_2_ S‐LiMn_0.85_Cr_0.15_O_2_	2.0–4.4 2.0–4.4	180/50 mA g^−1^ 144/50 mA g^−1^	169/93.9%/40 121/84%/40	[118] [118]
Y^3+^	0.090	LiMn_0.98_Y_0.02_O_2_	2.0–4.4	191/25 mA g^−1^	173/90.6%/20	[119]
Fe^3+^	0.055	LiMn_0.95_Fe_0.05_O_2_	2.0–4.5	200/–	≈180/90%/30	[96]
		LiMn_0.95_Fe_0.05_O_2_	2.0–4.3	≈60/22.5 mA g^−1^	≈68/113.3%/50	[120]
Ti^3+^	0.076	LiMn_0.95_Ti_0.05_O_2_	2.0–4.5	≈32/0.2 C	≈132/412.5%/60	[121]
B^3+^	0.023	m‐LiMn_0.95_B_0.05_O_2_	2.0–4.5	≈152/50 mA g^−1^	150/98.7%/100	[56]
Bi^3+^	0.096	Li_0.75_Mn_0.99_Bi_0.011_O_2_	2.0–4.3	242/60 mA g^−1^	≈175/72.3%/50	[94]
S^2−^	0.184	Li_0.56_MnO_1.98_S_0.02_	2.0–4.6	170/0.4 mA cm^−2^	220/117.6%/50	[122]
F^−^	0.136	Li_0.8_6MnO_1.98_F_0.02_	2.0–4.3	129/50 mA g^−1^	210/162.8%/50	[123]
BO_3_^3−^	–	Li_0.64_MnO_1.991_(BO_3_)_0.009_	2.0–4.3	≈209/60 mA g^−1^	≈163/78%/50	[94]
PO_4_^3−^	–	Li_0.67_MnO_1.993_(PO_3_)_0.007_	2.0–4.3	≈221/60 mA g^−1^	≈137/62%/50	[94]
SiO_3_^3−^	–	Li_0.65_MnO_1.988_(SiO_3_)_0.012_	2.0–4.3	≈173/60 mA g^−1^	≈162/93.6%/50	[94]

### 3.2 Surface Coating

Element doping is particularly effective in stabilizing the bulk structure, while coating makes great sense to protect the surface of electrode materials for resisting manganese dissolution into the electrolyte and impeding surface reactions.

Al_2_O_3_, an ionic and electronic insulation, is often used as a coating for the cathode to prevent active electrode materials from being corroded by electrolytes. Cho et al. confirmed that the capacity loss of the Al_2_O_3_‐coated o‐LiMnO_2_ (only 2% after 50 cycles) was significantly lower than that of the bare one (35% loss).^[^ ^135^ ^]^ Most significantly, there are no Li_2_Mn_2_O_4_ phases in the Al_2_O_3_‐coated electrode during the process of cycling compared with the uncoated one, implying that the protective coating layer can keep the lattice stable and suppress the J–T distortion. However, the LiMn_1‐_ *_x_* Al*_x_* O_2_ solid solution or LiAlO_2_ may be formed during the process of coating Al_2_O_3_ with sol‐gel and heating treatment.^[^ ^136^ ^]^ For the materials prepared at high temperatures (600 °C and 700 °C), the uniform formation of the LiMn_1‐_ *_x_* Al*_x_* O_2_ solid solution across the particle instead of staying on the surface may bring the rapid capacity decrease. The LiAlO_2_ peak can be observed from the XRD pattern, indicating that the Al_2_O_3_ gel solution would react with the o‐LiMnO_2_ on the surface of materials. Likewise, similar effects can be observed in CoO‐coated o‐LiMnO_2_.^[^ ^137^ ^]^ Even if CoO‐coated LiMnO_2_ exhibits a much lower initial discharge capacity (127 mAh g^−1^) compared to that of the bare one (162 mAh g^−1^), the former presents rapidly increasing capacity (185 mAh g^−1^ after 15–20 cycles) and just 12% capacity decay after 50 cycles. A significant amount of Co atoms appears in the range of 2‐µm range near the surface than in the bulk, which is a sign of solid solution formation on the surface. Thanks to the presence of solid solutions, the electrochemical stability of CoO‐coated LiMnO_2_ has been improved at 55 °C. In addition, the disappearance of the Li_2_MnO_3_ impurity phase after Al_2_O_3_/CoO coating implies that the coating layer lies on the surface of LiMnO_2._^[^ ^138^ ^]^ It may be a reference for the removal of Li_2_MnO_3_ impurity that occurred in the synthesis of related lithium‐manganese oxygen cathodes. There are many similar effects in Al_2_O_3_ and CoO‐coated o‐LiMnO_2_. However, only CoO‐coated LiMnO_2_ displays an additional voltage plateau at 2 V probably causing capacity decay. This phenomenon illustrates the structural disorder of Al_2_O_3_/CoO coating is different.

Lithium boron oxide (LBO) with good ionic conductivity has been successfully used to coat spinel and binary/ternary layered cathode materials for improving cycling stability. There is no doubt that above mentioned cycling stability is also observed in the LBO‐modified o‐LiMnO_2_. Nagasubramanian et al. studied the cycling performance of the LBO‐coated o‐LiMnO_2_ and its potential explanation.^[^ ^139^ ^]^ During the first charge‐discharge, the intercalation/deintercalation behavior of Li^+^ is considered to be irreversible, which can be attributed to the structure transformation from the layered to the spinel. But as the phase transition is completed during cycles, the maximum capacity is raised to 189 mAh g^−1^ and the electrode retains 91% of the maximum capacities after 70 cycles, while only 77% of capacity can be obtained for uncoated one. By observing the contribution of discharge capacity from different platforms (3 V and 4 V) in the charge/discharge curves before and after coating, it can be seen that the major capacity loss is occurring from the 3 V region. Coupled with significantly reduced charge‐transfer resistance in the corresponding voltage range, it is obvious that LBO is beneficial for Li^+^ inserting into the octahedral voids in the LiMn_2_O_4_ structure, resulting in less capacity loss.

**Figure 7:**
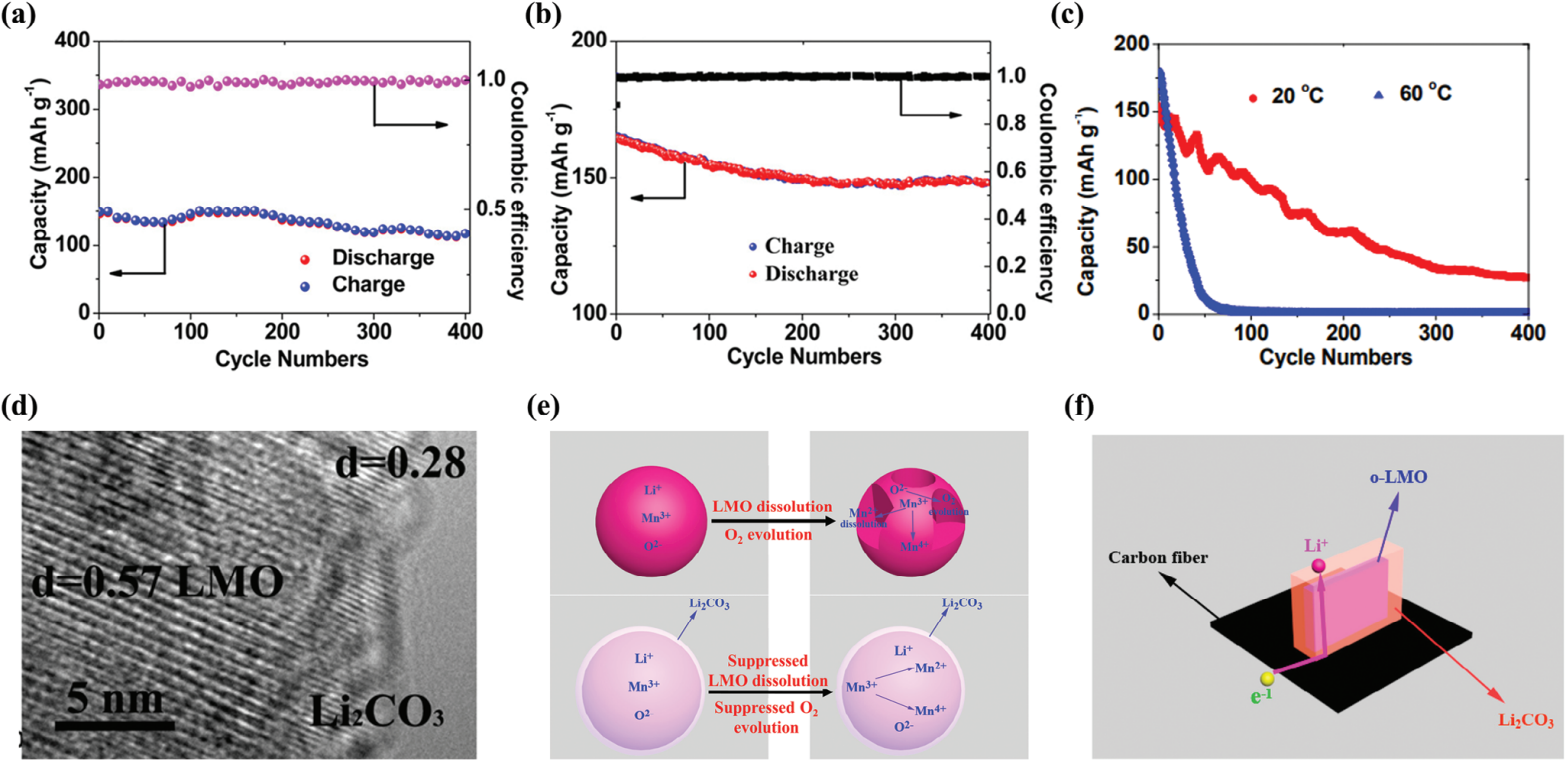
Cycling performance of the o‐LMO@Li_2_CO_3_ nanosheet array cathode at 2 C at a) 20 °C and b) 60 °C. c) Cycling performance of the uncoated o‐LMO cathode at 0.5 C. d) High‐resolution TEM image of o‐LMO@Li_2_CO_3_ nanosheet array electrode. e) Proposed interfacial change of the bare o‐LMO electrode and that coated with a Li_2_CO_3_ layer during charge/discharge process. f) Pathway of electron transport in the o‐LMO@Li_2_CO_3_ nanosheet array electrode. Reproduced with permission.^[^ ^141^ ^]^ Copyright 2016, American Chemical Society.

**Figure 8:**
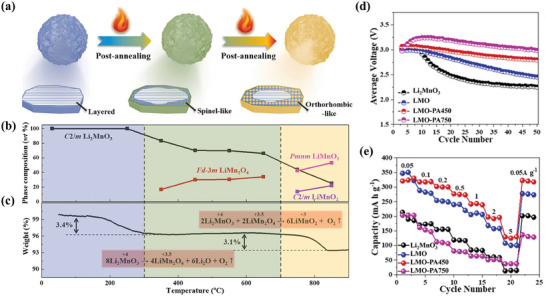
a) Schematic illustration of core‐shell structural architecture with the annealing treatment. b) The evolution of the phase composition with the temperature based on Rietveld refinements of the corresponding XRD patterns. c) TGA analysis of pristine LMO. d) Cycling stability at 25 °C and 50 mA g^−1^ and e) rate capability at a different current density from 10 to 1000 mA g^−1^ for the samples Li_2_MnO_3_, LMO, LMO‐PA450, and LMO‐PA750. Reproduced with permission.^[^ ^142^ ^]^ Copyright 2022, Elsevier.

In recent years, electrode materials with core‐shell structures have been aggressively developed to resolve the problem of low capacity and poor cyclic instability.^[^ ^140^ ^]^ Generally speaking, the shell can be formed by coating and acts as a protection layer to make better overall performance of the materials. Guo et al. confirm that designing an o‐LiMnO_2_@Li_2_CO_3_ nanosheet array cathode with a core‐shell structure presents better electrochemical behavior.^[^ ^141^ ^]^ Intriguingly, the o‐LiMnO_2_@Li_2_CO_3_ electrode shows 80% and 79% initial capacity after 400 cycles at 2C at 20 °C and 60 °C, respectively (**Figure** **7**a and 6b), yet the control electrode without Li_2_CO_3_ coating at 0.5 C only exhibits capacity retention of 18% at 20 °C and less than 1% at 60 °C after 400 cycles (Figure 7c). The reasons for the excellent cycling performance of o‐LiMnO_2_@Li_2_CO_3_ nanosheet array electrode can be explained in Figure 7d–f. First, the outer layer of Li_2_CO_3_ on the o‐LiMnO_2_ surface can be confirmed by high resolution transmission electron microscope (HRTEM) (Figure 7d). The outer layer can significantly minimize or avoid adverse reactions (o‐LMO dissolution and oxygen release) (Figure 7e). The Li_2_CO_3_ is one of the parts of the SEI layer, the Li_2_CO_3_ layer is beneficial to electrochemical stability at elevated temperatures. Second, the nanosheet array structure is not only equipped with a 1D pathway for fast electron transport but also shortens the diffusion length of lithium, which ensures sufficient contact of active material with electrolyte (Figure 7f). Huang et al. induced two different core‐shell microstructures of Li‐Mn‐O materials by heating treatment (**Figure** **8**a).^[^ ^142^ ^]^ The phase transformation is summarized in detail in Figure 8b, there are three stages at 300 °C and 700 °C, corresponding to two transformations. TGA analysis was provided in Figure 8c. When annealed to 350°C, the oxygen on the surface of Li_1.11_Mn_0.76_O_2_ (LMO) is released and the lithium diffuses to form Li_2_O. Thus, the lithium vacancies are occupied by Mn to form spinel, resulting in a layered core and spinel shell. As the annealing proceeds to higher temperatures, the internal Li^+^ diffuses to the external spinel, forming a LiMnO_2_ shell. That is, a LiMnO_2_ shell wrapped around a spinel core is formed at 750 °C. The cycling performance and rate capability of these two core‐shell structural materials are shown in Figure 8d,e. Notably, the annealing‐induced synthesis method ensures the homogeneity of the core‐shell structure. Compared with conventional coating methods, this approach presents atomic‐level contacts between the cores and shells.

In brief, modification by coating has resulted in a relatively feasible solution of optimizing the electrode surface to improve electrochemical performance. For instance, the coating layer reduces phase transformation (from the layer to spinel), decreases capacity loss, facilitates Li^+^ transfer, and increases capacities. Particularly, the coating delivers high capacity retention even at elevated temperatures, which may be attributed to impeding the active materials touch with the electrolyte solution, thus the side effects of LiMnO_2_ are minimized during cycling, especially manganese dissolution. Maybe coating is just like eggshells, keeping the active material safe in its role. But how to get the depth of the coating to hit the spot in the process of material synthesis still needs further study. Additionally, research on LiMnO_2_ coating appears to be slightly less than other cathodes (such as LiCoO_2_ and LiMn_2_O_4_). Co‐coating can be considered as a new modification method to improve the electrochemical performance of LiMnO_2_, due to its synergistic effect.

### 3.3 Composites Designing

To break through the bottleneck of imperfect LiMnO_2_ cathode, introducing other favorable materials to fabricate composites has been investigated in recent years. These materials are conducive to the electrochemical performance of electrodes, especially for carbon materials. LiMnO_2_‐carbon composites are usually prepared by mixing carbon materials (carbon nanotubes (CNTs), reduced graphene oxide (rGO) and graphene nanoplatelet (GNP)) with prepared LiMnO_2_ or with raw material for the preparation of LiMnO_2_. The carbon materials in LiMnO_2_‐carbon composites can offer high electronic conductivity and fast Li^+^ diffusion, which contributes to enhanced specific capacity, improved capacity retention and reduced electrochemical impedance relative to pristine LiMnO_2_.^[^ ^143^, ^144^ ^]^

**Figure 9:**
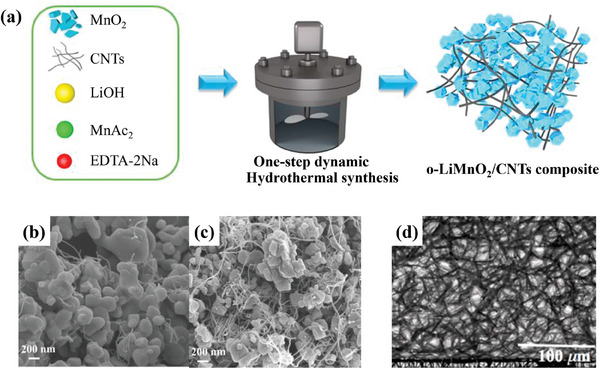
a) One‐step dynamic hydrothermal synthesis of o‐LiMnO_2_/CNTs composites. SEM images of b) 1%CNT‐LMO, c) 5%CNT‐LMO with one‐step dynamic hydrothermal synthesis. Reproduced with permission.^[^ ^145^ ^]^ Copyright 2021, Elsevier. d) SEM image of o‐LiMnO_2_‐MWCNTs. Reproduced with permission.^[^ ^146^ ^]^ Copyright 2019, Elsevier.

**Figure** **9** is the scheme and scanning electron microscope (SEM) images of orthorhombic LiMnO_2_/CNTs nanocomposites.^[^ ^145^, ^146^ ^]^ As schematically illustrated in Figure 9a, o‐LiMnO_2_/CNTs composites were prepared by a one‐step dynamic hydrothermal way. And the corresponding SEM images show that LiMnO_2_ particles are “confined” among CNTs (Figure 9b,c).

Figure 9a is the prepared scheme of o‐LiMnO_2_/CNTs composites through a one‐step dynamic hydrothermal way, and the corresponding SEM images show that LiMnO_2_ particles are “confined” among CNTs (Figure 9b,c). The composite with 5 wt% CNTs brings about the optimum specific capacity of 204.9 mAh g^−1^. The o‐LiMnO_2_‐MWCNTs nanocomposites, where CNTs instead of carbon black worked as an additive material, display the intricate network in Figure 9d. The addition of CNTs reduces the charge transfer resistance from 190 to 105 kΩ. The 3D network provided by CNTs increases electronic conductivity of the samples and facilitates the Li^+^ diffusion across the interface of electrolyte. Tian et al. synthesized LiMnO_2_@rGO composites via a one‐pot hydrothermal route at 200 °C for 12 h,^[^ ^147^ ^]^ in which LiMnO_2_ nanoparticles were uniformly anchored onto rGO nanosheets. It is rewarding to note that the rGO with superior conductivity provides a stable conductive net, thus the first discharge capacity of LiMnO_2_@rGO could be improved to 175 mAh g^−1^, as well as capacity retention increased from 53.53% to 80.97% after 100 cycles.

**Figure 10:**
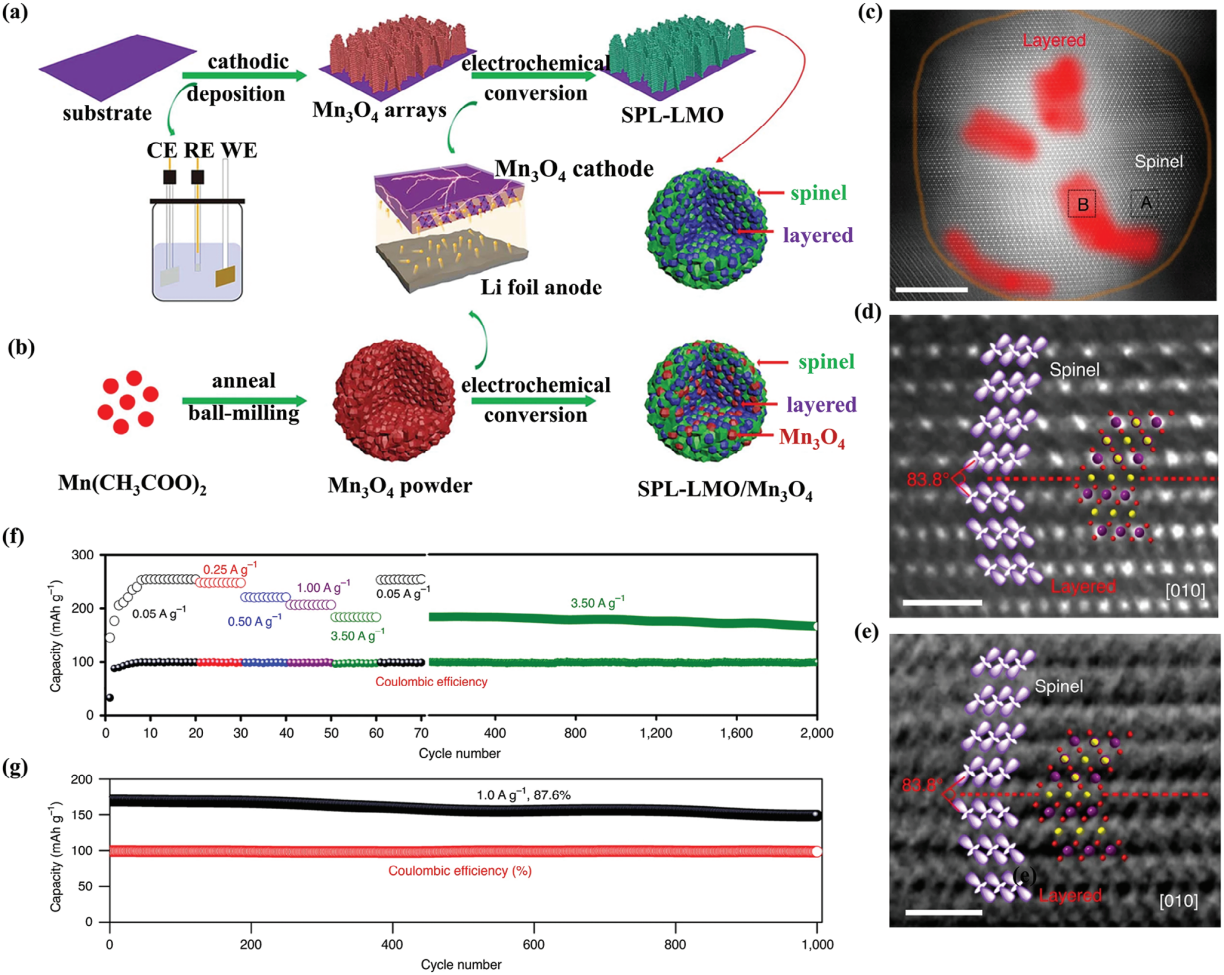
a) Synthesis of SPL‐LMO (CE, counter electrode; RE, reference electrode; WE, working electrode). b) Synthesis of SPL‐LMO/Mn_3_O_4_. c) HAADF‐STEM image for the SPL‐LMO nanoparticle. Scale bar, 5 nm. d) HAADF‐STEM image and e) ABF‐STEM image at the spinel‐layered interface for the SPL‐LMO sample along the [010] zone axis. Scale bar, 1 nm. f) Rate capability and cycle performance of the SPL‐LMO cathode. g) Cycle performance of the SPL‐LMO//graphite full cell. Reproduced with permission.^[^ ^148^ ^]^ Copyright 2021, Springer Nature.

Although manganese‐based cathode materials are of great significance for the sustainability of LIBs, the J–T distortion of Mn^3+^ has become a bottleneck to improving their structural stability. Zhu et al first proposed a nice idea to suppress the J–T distortion of Mn^3+^ using interfacial orbital ordering.^[^ ^148^ ^]^ As shown in **Figure** **10**a,b, the in situ electrochemical conversion from Mn_3_O_4_ was used to prepare heterostructured spinel‐layered LiMnO_2_ (SPL‐LMO). Layered domains within the spinel matrix and the orientation of orbitals at the spinel‐layered interface can be seen in Figure 10c–e, where MnO_6_ of the layered and spinel phases is linked with an included angle of 83.8°. Significantly, the SPL‐LMO materials exhibit superior rate performance (Figure 10f) as well as cycling stability of the full cell at a current density of 1.0 A g^−1^ for 1000 cycles (Figure 10g), which is superior to layered or spinel LiMnO_2_ cathodes reported previously.

### 3.4 Compatible Electrolyte

The electrolyte is one of the most important components of the battery, as it endows with the ability to establish ionic conductive channels and block the electronic conductivity between the cathode and anode. The development of electrolytes for LiMnO_2_ has been underway for several years.

In 1997, the group of Davidson researched the electrochemistry of LiMnO_2_ over the range of 1.0‐4.6 V with a variety of electrolytes.^[^ ^149^ ^]^ Li/LiMnO_2_ cells exhibit a higher discharge capacity in 1 M LiClO_4_ ethylene carbonate (EC)/dimethyl carbonate (DMC) electrolyte (257 mAh g^−1^) than that of 1 M LiPF_6_ EC/DMC electrolyte (220 mAh g^−1^) between 1.0 and 4.4 V. In addition, the voltage range also has a significant effect on the charge and discharge capacity. For example, with 1 M LiClO_4_ in propylene carbonate (PC)/EC, charge and discharge capacities are 253 and 250 mAh g^−1^ over the range of 1.0–4.4 V, respectively. But when charging to 4.2 V, it displays only the charge capacity of 228 mAh g^−1^. However, they give no further explanation. Thus, substantial differences occur when varying the electrolytes, which also needs to adjust the voltage range for better compatibility.

Based on 1, 3‐dioxolane‐LiAsF_6_ solutions, Tadiran developed the LiMnO_2_ rechargeable battery with excellent electrochemical performance.^[^ ^150^ ^]^ In 1, 3‐dioxolane‐LiAsF_6_ solutions, the surface film consisting of Li alkoxides, Li formate, LiF and Li*_x_* AsF*_y_* is formed on the Li anode, which induces uniform and smooth Li deposition. Thus, the smooth morphology of Li deposition provides a high cycle life.

The layered lithium transition metal oxides tend to release oxygen in a highly delithiated state, possibly accompanied by an exothermic reaction with the electrolyte. These could be the most serious threat to the safety of batteries. Reassuringly, when o‐LiMnO_2_ cathode contacts with 1 M LiPF_6_ solution in 1:1:1 wt.% EC/diethyl carbonate (DEC)/DMC solvent, DSC measurements show a much lower heat effect during the first process compared with other cathode materials.^[^ ^151^ ^]^

Solid polymer electrolyte (SPE) has the advantage of excellent processability and flexible tunability. All of these advantages make it particularly valuable in designing and developing high‐energy, long‐life solid state lithium batteries. Lithium polymer batteries (LPBs) involved LiMnO_2_ cathode have been investigated in recent years, which relates to SPE such as polyethylene oxide (PEO), polyvinylidene fluoride‐hexafluoropropylene (PVDF‐HFP), and polyvinylidene fluoride (PVDF). Xia et al. evaluated the thermal stability of LiMn_2_O_4_ and LiMnO_2_ in PEO polymer electrolyte. DSC curves show that delithiated LiMn_2_O_4_ decomposes at 230 °C, while the decomposition of LiMnO_2_ with partial charge occurs at 350 °C. It is worth noting that the LiMnO_2_ cathode displays much higher thermal stability than that of LiMn_2_O_4_ when the PEO polymer is used as an electrolyte.^[^ ^152^ ^]^ Gu's group obtained PVDF‐HFP‐PC_10_EC_10_LiClO_4_ as SPE to fabricate LiMnO_2_/SPE/Li battery. First, they prepared a mixed solution consisting of LiClO_4_, EC, PC, and 25 wt.% PVDF, and then casting and quickly heating it at 110 °C for 10 min.^[^ ^153^ ^]^ Compared with LiMnO_2_/Li battery with the 1 M LiPF_6_ in EC/DMC (EC:DMC = 1:1) liquid electrolyte, LiMnO_2_/SPE/Li battery shows a greater discharge capacity of 124 mAh g^−1^.^[^ ^154^ ^]^ In addition, LiMnO_2_‐PAn‐DMcT composite cathode was prepared by mixing LiMnO_2_ powder with polyaniline (PAn), 2,5‐dimercapto‐1,3,4‐thiadiazole (DMcT) and carbon in N‐mehtylpyrrolidinone (NMP).^[^ ^155^ ^]^ LiMnO_2_‐PAn‐DMcT composite cathode can increase the capacity of lithium polymer battery. In addition, PVDF‐based SPE has been intensively studied due to its high mechanical strength and good thermal stability. In 2021, Fouladvand et al. prepared a PVDF/SGO polymer electrolyte by introducing sulfonated graphene oxide (SGO) as an effective nanofiller. LiMnO_2_ cathode matched with PVDF/SGO polymer electrolyte obtains a high discharge capacity of 204 mAh g^−1^ at 0.1 C.^[^ ^156^ ^]^

All solid‐state batteries (ASSBs) are the strong contender for the new energy industry due to their longer cycle life, higher energy density and safety compared to conventional liquid batteries. And further research into competitive high energy‐density ASSB is focused on small‐size cathode composite material consisting of active material, solid state electrolyte (SSE) and conductive additive. In order to enhance the charge transfer of Li^+^, active material must bond strongly with SSE by high temperature annealing. Rumpel et al. investigated the thermal stabilities of LiMnO_2_ and LiMnPO_4_ (LMP) in ASSB, which was assembled with the ceramic electrolyte Li_1.3_Al_0.3_Ti_1.7_(PO_4_)_3_ (LATP).^[^ ^157^ ^]^ Unfortunately, LiMnO_2_ decomposes into Mn_3_O_4_ and LiMn_2_O_4_ at <500 °C because of the disproportionation of Mn^3+^. In contrast, LATP remains thermally stable even under 800 °C in an argon atmosphere.

In conclusion, LiMnO_2_ as a cathode can be used in various batteries, including non‐aqueous batteries, LPBs, and ASSBs. To facilitate the commercialization of LiMnO_2_ cathode, the design of electrolytes is of great significance. Thus, the application of LiMnO_2_ cathode can be researched in more fields.

## 4 Conclusion and Outlook

It has been 30 years since the commercialization of LIBs in 1991, and seeking for electrodes of low cost, high energy density, high safety and long life is always the primary aim to develop new‐generation LIBs in the future.

Facing resource depletion and the quest for non‐toxic green resources, lithium manganese oxide as a cathode material shows highly favorable advantages. Previous studies have shown that elemental doping is a good way to keep the LiMnO_2_ layers in place by suppressing phase transitions caused by the J–T effect due to high spin Mn^3+^. But it is not even close to commercialization because the charge/discharge mostly remains ≈50 cycles, especially for onefold doping. For another thing, surface coating is another effective approach to resolve the Mn dissolution and improve the cycling stability of LiMnO_2_, however, it also suffers from the complex procedures during coating and electrochemical‐inert coating layers that decrease the capacity of LiMnO_2_.

Thus, further efforts are still required to address the key issues and make commercialization achievable for LiMnO_2_. Special attention needs to be paid to the following aspects such as design of Li‐rich lithium manganese oxides, multi‐functional coating layers and new liquid electrolytes to achieve higher energy and more stable performance. Meanwhile, from the viewpoint of practical application with high sustainability, all‐solid‐state batteries systems with LiMnO_2_ as cathodes should be further exploited and the repairing/recycling techniques of spent LiMnO_2_ are necessary to carry forward.

1) In the LiTMO_2_ family, Li‐rich lithium manganese oxides (like Li_1+_ *_x_* TM_1‐_ *_x_* O_2_) with the disorder‐rocksalt structure are a class of cathode materials that can deliver ultrahigh capacity (> 300 mAh g^−1^) and energy density (>1000 Wh kg^−1^). It is aspired that effective methods could be developed for the controlled preparation of Li‐rich lithium manganese oxides to meet the request in the area of high‐energy applications.
2) It is highly desirable to construct multi‐functional coating layers with excellent mechanical stability to avoid Mn dissolution and side reactions, electrochemical activity to contribute capacity and electronic/ionic conductivity to facilitate charge transfer, which needs to design composite coating layers with elaborate element combinations other than traditional ones.
3) To reduce side reactions of LiMnO_2_ with electrolyte solution and Mn dissolution at most, the design and development of new electrolytes matching LiMnO_2_ cathodes is also a good solution.
4) All‐solid‐state batteries feature high energy density and high safety. LiMnO_2_‐based all‐solid‐state batteries are deemed to eliminate the problem of Mn dissolution and to deliver higher energy density. However, LiMnO_2_‐based all‐solid‐state batteries have been seldomly reported, which should be paid special attention to and developed as a priority for purpose of practical applications.
5) The resource shortage of lithium has raised the price of raw materials of cathode materials as well as the cost of batteries. Therefore, to address the concern in cost as well as the environment, the repairing and recycling techniques are urgent for spent LiMnO_2_ cathode materials.

## Conflict of Interest

The authors declare no conflict of interest.
